# Perceptions of Pharmacy Students on the E-Learning Strategies Adopted during the COVID-19 Pandemic: A Systematic Review

**DOI:** 10.3390/pharmacy10010031

**Published:** 2022-02-15

**Authors:** Carla Pires

**Affiliations:** CBIOS—Universidade Lusófona’s Research Center for Biosciences and Health Technologies, 1749-024 Lisbon, Portugal; p5558@ulusofona.pt

**Keywords:** COVID-19, e-learning, online education, pharmacy students, perception, opinion

## Abstract

Background: E-learning strategies were globally adopted by academies because of the COVID-19 pandemic. The characterization of students’ perception of online learning is fundamental to design appropriate models for pharmacy curricula. The study aim was to carry out a systematic review about the perception of pharmacy students on the e-learning strategies adopted during the COVID-19 pandemic. Methods: The Preferred Reporting Items for Systematic Reviews and Meta-Analyses (PRISMA) checklist was followed. PICOS criteria were applied. Five databases were screened: PubMed, Cochrane Library, DOAJ—Directory of Open Access Journals, SciELO—Scientific Electronic Library Online and b-on—Online Library of knowledge (Biblioteca do conhecimento online). Keywords: “pharmacy and (distant or remote or e-learning or online or zoom or education or training or digital) and (COVID or SARS and (student or undergraduate) and (opinion or satisfaction or perception or attitude)”. Results: 23 out of 176 papers were selected (28 duplicated and 125 excluded). Selected papers were classified, as follows: studies exclusively involving pharmacy students (n = 8); studies simultaneously involving pharmacy students and other healthcare students (n = 6); and studies related to the involvement of pharmacy students in specific courses (n = 9). Conclusions: In general, the perception of pharmacy students on e-learning strategies adopted during the COVID-19 pandemic was positive. However, an expressive proportion of undergraduates reported negative issues about online education, which seems to support the necessity of optimizing e-learning strategies in the future.

## 1. Introduction

E-learning or online-learning (i.e., any students’ online learning activity, with the involvement of digital technologies) and e-teaching strategies (i.e., any online teaching activity, with the involvement of digital technologies) were globally adopted by schools and academies during the COVID-19 pandemic. This shift to online education was quickly implemented as a mitigation measure of the propagation of SARS-CoV-2. Lockdowns and confinements required diverse transitions/adaptations from in-person classes to online activities/classes, such as theoretical or experiential training, students’ assessments, extracurricular activities, or student support [1]. Health courses were not an exception, regarding the adoption of new online curricular formats [2,3]. Usually, both synchronous and asynchronous components were adopted in e-learning classes [3].

Before the pandemic, in-person classes were the predominant practice, with e-learning only constituting a complementary teaching alternative in most of the developed countries (e.g., through Moodle, Zoom or other online platforms). [4,5]. However, the use of online teaching exponentially increased after the beginning of the pandemic, including in developing countries. Globally, diverse limitations were identified regarding medical and pharmacy online education, such as lack of equipment, the high costs of Internet bundles, or difficulties in providing practical and clinical teaching. The adoption of e-learning was difficult in some countries due to a lack of internet access, such as Africa, where internet coverage is limited [2,3,6].

The advantages of e-learning are described in diverse studies, such as, time flexibility, easy administration, accessibility, comfort, self-directed learning, and asynchronous learning [7,8]. Among other things, students identified the following benefits of e-learning. Some examples of text transcripts from students are then presented: “Easier to get to the lecture on time”, “I have more time to study and finish assignments now”, “It’s great to study from home, and I have time to write all the notes I need which helps me keep up with the curriculum”, or “The positive points to me are that we can review our lecture more than once and at any time” [7]. On the contrary, diverse weaknesses of online education were also described, such as lack of contact with the instructor/teacher, lack of human interactions, lack of immediate feedback, inadequate internet connectivity, technical issues, or more limited interactions with colleagues [7,9]. Additionally, the substitution of in-person laboratorial or clinical practices by online education presented some constrains. The real impact of e-learning on the practical clinical skills of health students is not yet known [10].

The potential impact of online education on the students’ mental health is one of the worst disadvantages. For instance, several pharmacy students reported isolation and mental health issues in a thematic qualitative analyze, such as “We are socially isolated. Try to create virtual event where we can meet other students, that we actually want to go to” or “The fall semester took a bit toll on my mental health and I’m sure I’m not the only one who feels that way” [11]. In another study, 63.5% of the pharmacy students presented academic burnout, 44.6% reported exhaustion and 41.7% declared cynicism during the confinement [12].

Diverse studies state that e-learning may become the new norm in the future [13,14]. In general, health students from medicine and dentistry, nursing and health science showed a positive opinion about e-learning regarding their perceptions, acceptance, motivation, and engagement [2]. For instance, 56% of pharmacy students declared that they preferred the online delivery of courses over in-person classes in a study, which was carried before the COVID-19 pandemic. In this study, the online delivery format was a key factor of students’ choice of an elective course [15].

However, diverse methodological issues were identified in the design of studies on the evaluation of health students’ perceptions during the COVID-19 pandemic, such as the use of heterogeneous measurement instruments, the lack of a certainty assessment, and/or the lack of quality in the design of studies or uncertain reporting. These constraints may lead to limitations in the interpretation of study findings [2,3]. The lack of evidence of health students’ motivation and/or engagement with e-learning during the COVID-19 pandemic were other identified gaps [2]. Moreover, assessed students’ reaction/satisfaction, and/or assessed changes in attitudes, knowledge, or skills were heterogeneously evaluated [3]. Future research on the present topic must be methodologically rigorous [2].

Thus, comprehension of pharmacy students’ perceptions/opinions on e-learning during COVID-19 pandemic is relevant to design a new educational model. In this sense, the study aim was to carry out a systematic review about the perception of pharmacy students on the e-learning strategies adopted during the COVID-19 pandemic.

## 2. Materials and Methods

### 2.1. Previously Identified Systematic Reviews on the Same and/or Similar Topics

Overall, systematic reviews specifically about the perceptions of pharmacy students regarding e-learning during COVID-19 were not found in the screened databases (PubMed, SciELO, b-on, DOAJ, and Cochrane Library) [16,17,18,19,20]. For instance, no review was identified in Cochrane Library on 16 January 2022), with the keywords “pharmacy and student and COVID” or “pharmacy and student and SARS”. These findings support the relevance of the present systematic review to the state of the art on the present topic.

However, one systematic review on the perceptions of health students on online education during the COVID-19 pandemic was identified. It concluded that health students presented a positive response, regarding e-learning perceptions, acceptance, motivation, and engagement. This systematic review covered diverse types of health students, as follows: medical, dental, nursing, and health science students, but not pharmacy students [2].

### 2.2. Concepts and Definitions

In the present study, e-learning or online-learning should be understood as any students’ online learning activity, with the involvement of digital technologies and e-teaching strategies should be understood as any online teaching activity, with the involvement of digital technologies [1].

Studies related to pharmacy students’ opinions, levels of satisfaction, perceptions, or attitudes towards or with e-learning during the COVID-19 pandemic (study intervention) were included in the present systematic review. A brief definition of these concepts is presented, as follows: perceptions [21] (i.e., *an idea, a belief or an image you have as a result of how you see or understand something*), satisfaction [22] (i.e., *the good feeling that you have when you have achieved something or when something that you wanted to happen does happen; something that gives you this feeling*), attitude [23] (i.e., *the way that you think and feel about somebody/something; the way that you behave towards somebody/something that shows how you think and feel*), and/or opinions [24] (*your feelings or thoughts about somebody/something, rather than a fact*), regarding e-learning during the COVID-19 pandemic.

### 2.3. Responsible for the Collection and Analysis of Selected Papers

Only the study author was responsible for the collection and analysis of the selected papers. All results were double checked. All searches and analyses were documented and archived for further evaluation. All steps and methodologies of the present systematic review are described in the present paper.

### 2.4. Preferred Reporting Items for Systematic Reviews and Meta-Analyses (PRISMA) and PICOS

The PRISMA checklist and flow diagram were followed [25,26]. PICOS (P:population; I: intervention; C: comparisons; O: outcomes; S: study design) criteria were applied (Table 1) [27].

### 2.5. Inclusion and Exclusion Criteria

All studies (quantitative or qualitative) involved the collection of pharmacy students’ opinions, levels of satisfaction, perceptions, or attitudes to e-learning during the COVID-19 pandemic (inclusion criteria). Reviews (narrative, systematic or meta-analysis), commentaries, repeated studies, grey literature, and studies written in other languages than English, Portuguese, Spanish, French or Italian were excluded (exclusion criteria).

### 2.6. Screened Databases and Timeframe

Overall, five databases (PubMed, Cochrane Library, DOAJ–Directory of Open Access Journals, SciELO–Scientific Electronic Library Online and b-on–Online Library of knowledge (*Biblioteca do conhecimento online*)) [16,17,18,19,20] were conveniently selected because they comprise a large number of high-quality peer reviewed papers. For instance, Google Scholar was not selected, because this database cover non-peer-reviewed papers/works. The Cochrane Library was screened to identify potentially relevant and highly quality reviews on the same or similar topics [20].

All databases were screened during January 2022, as follows: PubMed (7 January 2022); Cochrane Library (16 January 2022), DOAJ (10 January 2022), SciELO (16 January 2022), and b-on (14 January 2022) [16,17,18,19,20]. Only papers published after the beginning of the COVID-19 pandemic were included (i.e., after 11 March 2020).

### 2.7. Keywords

The string of the selected keywords was as follows: “pharmacy and (distant or remote or e-learning or online or zoom or education or training or digital) and (COVIDor SARS and (student or undergraduate) and (opinion or satisfaction or perception or attitude)”. Diverse synonyms, and related terms of different keywords were browsed to maximize the number of potentially relevant search terms, as recommended in the guidelines about the selection/definition of keywords. [28].

## 3. Results

### 3.1. Selected Studies

**Figure 1:**
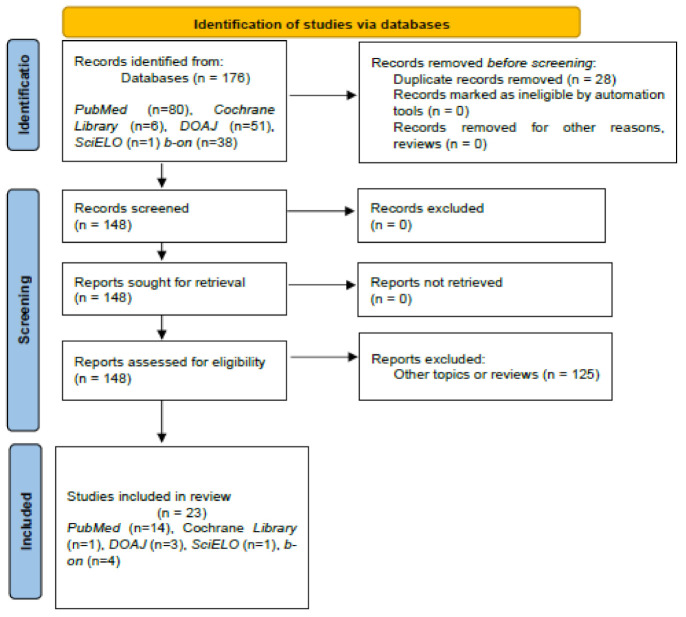
Preferred Reporting Items for Systematic Reviews and Meta-Analyses (PRISMA) 2020 Flow Diagram for new systematic: pharmacy students’ perceptions on e-learning during the COVID-19 pandemic [25,26].

Twenty-three papers were selected, which were distributed, as follows: PubMed (n = 14), Cochrane Library (n = 1), DOAJ (n = 3), SciELO (n = 1) b-on (n = 4) (Figure 1). Overall, 22 out of the 23 selected papers were written in English and 1 paper was written in Portuguese.

### 3.2. Main Findings of Selected Studies

The main findings of the 23 selected studies are presented in Table 1. The selected studies were classified, as follows:

- Studies exclusively involving pharmacy students (n = 8);
- Studies simultaneously involving pharmacy students and other healthcare students (n = 6); and
- Studies related to the involvement of pharmacy students in specific courses/ activities (n = 9).

These three categories were conveniently selected based on a qualitative analysis of the content of the 23 selected papers.

Saudi Arabia was the country with most publications (5 out 23), followed by Australia (3 out 23) and Jordan, China, USA, Canada, or United Kingdom (2 out 23). The countries with just one publication were, Canada, United Arab Emirates, Brazil, Sri Lanka, Spain, Sultanate of Oman, Malaysia, Estonia. Around 5000 pharmacy students have participated in the 23 selected studies (Table 2).

#### 3.2.1. Studies Exclusively Involving Pharmacy Students

In general, only about half of the pharmacy students (in some cases slightly more than half, and in other cases slightly less than half) classified the online education as positive as face-to-face learning and declared that they preferred in-person classes over online classes [29,31,32,33]. Pharmacy students declared to learn more in face-to-face learning than online classes in some studies [29,31]. Challenges of e-learning were predominant over benefits in diverse studies [35,36]. Thus, online classes for pharmacy students should be optimized in the future. 

Diverse challenges and/or difficulties have been identified regarding e-learning, such as the appearance of health issues due to long-time screen use, less communication in relation to face-to-face learning, a negative emotional response (frustration and anxiety were frequently reported), a negative impact on their education (e.g., less knowledge acquisition or it takes longer to get through material) or a distracting home environment [34,35,36]. In contrast, diverse benefits were identified, such as more comfort, less time spent travelling, more family time, and feeling valued and helpful during the pandemic [34,35]. Some studies were not convergent, regarding some topics. For instance, the number of interactions with instructors and classmates were classified as appropriate in one study, but not in another one [34,35].

#### 3.2.2. Studies Simultaneously Involving Pharmacy Students and Other Healthcare Students

In general, the other healthcare students, such as students from medicine or nursing or students from other general sciences, also reported diverse limitations (around half of the students), regarding their perceptions on e-learning during the COVID-19 pandemic [37,38,40,42]. All health students were favorable to the use of WhatsApp, Blackboard platform and the application of a HyFlex System, which was connected in face-to-face mode, online, or a mixture of both [39,41,42].

#### 3.2.3. Studies Related to the Involvement of Pharmacy Students in Specific Courses/Activities

Similarly, only around half of the pharmacy students (or a slightly more than half of the students in some cases) manifested positive perceptions on specific online courses during the COVID-19 pandemic (e.g., an anatomy and histology course; a virtual microbiology simulation; chemistry crossword puzzles as revision aids) [7,44,45,46]. Moreover, students’ understanding may have been affected in one study [7], and knowledge acquisition was not statistically different between both settings (i.e., in-person vs. online education) in another study [43]. Pharmacy students expressed positive perceptions of an online communication course, which may have been influenced by students previous e-learning experience [47]. OSCE courses seems to be feasible and easily implemented. Students’ perceptions were positive about this type of courses [6,48,49].

## 4. Discussion

The selected studies were not fully conclusive about students’ perceptions (i.e., positive, or negative) on adopted e-learning strategies during the COVID-19 pandemic. In general, only around half of health students classified as positive the adopted e-learning strategies during COVID-19 in the selected studies.

### 4.1. Studies Exclusively Involving Pharmacy Students

Overall, online education seems to be an acceptable option, which is likely to be more frequently offered in the future. However, e-learning strategies need to be optimized since only around half of pharmacy students had a positive perception of online education in the selected studies [29,31,32,33]. Students of the most advanced academic years manifested more favorable perceptions of e-learning than the students of the first year [33], but further studies are recommended to check this pattern.

A systematic review on healthcare students’ perceptions of e-learning during COVID-19 (medical and dental students, nursing, and health science students), reported positive perceptions in 7 out of 12 studies [2]. Among the possible improvements and optimizations of e-learning strategies are the adoption of proactive learning strategies, with more interactions with teachers and colleagues or better models for providing practical classes, an appropriate internet connectivity, clear instructions to carry out assessments, or a fair number of assessments [30]. Additionally, a diversified offer of e-learning options may be beneficial, such as lectures, tutorials, workshops, conferences, journal clubs, online sessions, or combinations of these [50,51].

It is highly recommended that pharmacy schools collect students’ perceptions/opinions through both quantitative and qualitative tools (e.g., Likert Scales plus open-ended questions). More qualitative studies are recommended to collect and characterize students’ perceptions on e-learning. Ideally, more details/information should be collected in these studies. Online education should be regularly supervised and optimized.

### 4.2. Studies Simultaneously Involving Pharmacy Students and Other Healthcare Students

Comparable results were obtained for the other healthcare students (e.g., medicine or nursing), with only about half of these students reporting a positive perception of the adopted e-learning strategies during the COVID-19 pandemic. Questionnaires seem to be useful tools to collect pharmacy students’ perceptions on e-learning strategies. This seems to confirm the need to optimize online education in diverse health areas. Online education affected health students’ social and psychological wellbeing, assessments, quality of education, or the administration of practical classes [37,38,40].

Social media platforms such as Facebook, Twitter, Instagram, YouTube, WhatsApp, podcasts, or the Blackboard Collaborate Ultra’s virtual showed to be powerful communication tools in interactive lecturing in medical education [52,53]. In this sense, online education through social media platforms is likely to improve pharmacy students’ satisfaction. However, there was a lack of guidance on the use of social media tools in online education [53] Thus, the development of international and national guidance on e-learning strategies for health students are recommended.

### 4.3. Studies Related to the Involvement of Pharmacy Students in Specific Courses/Activities

In general, students showed a positive perception of online education in the evaluated specific courses/activities. However, only around half of the pharmacy students presented a positive perception of the adopted e-learning strategies [6]. Students declared to prefer in-person over online activities, although they agreed with the offer of remote health system activities in the future [43].

Online pharmaceutical activities (e.g., online consultations) increased during COVID-19 [54,55]. Thus, online OSCE courses may be particularly relevant to simulate e-learning activities [6,48,49]. Additionally, hospitals and/or community pharmacies are likely to improve and increase the offer of this type of service in the future. It seems -learning strategies require optimization in the future (e.g., online OSCE courses).

Pharmacy schools need to supervise the administration of online courses aiming at updating and adjusting the new cycle of courses (if applicable). For instance, through the administration of questionnaires comprising both close-ended and open-ended questions (pre- and post- online courses). The previous e-learning experience of students should be evaluated since few studies carried out this assessment.

### 4.4. Limitations of the Selected Studies, Practical Implications and Future Research

Questionnaires seem to be an appropriate and suitable tool to evaluate pharmacy students’ perceptions of e-learning. Questionnaires/surveys were developed to evaluate the attitudes of pharmacy students based on different types of questions/topics, such as: “what do think about your attitude toward e-learning”, “I would prefer to have online learning to become the new normal”, “I feel comfortable to actively communicate with my classmates and instructors online”, “I feel that taking courses online will help me to remember/master them better”, “I prefer in-class approach as it provides a lot of interaction with my instructors and students” or “Distance learning has increased my collaborative work with my colleagues, whether in the dormitory or remotely” [7,33,50,51]. The questionnaires/questions were heterogeneously developed. Thus, comparisons across studies may be problematic/complicated. For instance, response rates were not described in all the studies. Ideally, standardized questionnaires and/or methodologies should be developed to evaluate pharmacy students’ perceptions of e-learning. These questionnaires should comprise both open-ended and close-ended questions. An international standard on perception questionnaires for pharmacy students should be developed in the future. The application of similar or equal questions and/or methodologies of administration (e.g., time or format) will facilitate comparisons across studies. 

More qualitative studies are recommended to characterize the perceptions of pharmacy students on e-learning. Pharmacy students’ perceptions of e-learning classes should be regularly collected since this variable was only identified in some of the selected studies. The necessary adjustments/adaptations of online classes should be implemented (if necessary/applicable) [56]. The number of qualitative studies was limited and did not follow the EQUATOR guidelines [57]. The previous e-learning experience of pharmacy students was only evaluated/reported in some studies.

Considering that the impact of COVID-19 pandemic may have influenced pharmacy students’ perceptions of e-learning (positively or negatively), long-term, multicentric, or longitudinal studies are recommended. New guidance on the present topic should address the main limitations of online education. Diverse optimizations of online classes are suggested, such as more interactions between teachers and students or between colleagues, more proactive learning strategies, new models for practical classes, clear instruction to carry out assessments or other practical activities and a fair number of assessments. Pharmacy students from more advanced academic years may have a more positive perception of e-learning than the first-year pharmacy students [33,38]. Besides pharmacy students’ perceptions, it is essential to study the effectiveness of online courses, such as students’ understanding. For instance, knowledge acquisition was only evaluated in some studies.

Further studies on the optimization of e-learning strategies are recommended, such as controlled studies comparing common vs. optimized e-learning methodologies, or prospective and multicentric studies to evaluate the impact of improved e-learning approaches. Successive questionnaires to collect students’ opinion, students’ interviews or both may be used to improve the current e-learning methodologies. The previous e-learning experience of students and response rates should be evaluated in future studies. The impact of e-learning on practical training/classes (e.g., chemistry) or mental health should also be supervised. Findings from these studies may be shared by different schools/colleges of pharmacy.

### 4.5. Study Limitations

Considering that selected studies were analyzed by just one author, consensus techniques were not applied to solve eventual divergencies between different researchers. A limited number of keywords and databases were used in the present systematic review. For instance, more synonyms of keywords or databases, such as Google Scholar could have been used. Thus, relevant studies on the present topic may have not been identified (e.g., studies from grey literature were not selected).

## 5. Conclusions

The number of selected studies on pharmacy students’ perceptions of e-learning strategies during COVID-19 pandemic was limited. Only about half of the sampled pharmacy students showed positive perceptions of online education. Online OSCE courses seem to be feasible and easily implemented, with pharmacy students expressing a positive perception of these courses. OSCE courses may be particularly relevant for training digital health skills, such as online pharmaceutical consultations. In general, the adopted e-learning strategies and/or online courses need to be optimized in the future. National or international guidance on the development and implementation of e-learning strategies for pharmacy students seems to be lacking.

## Figures and Tables

**Table 1:** PICOS definitions.

PICOS	Definition
Population (P)	Pharmacy students.
Intervention (I)	Any study that collects pharmacy students’ opinion, satisfaction, perception, or attitude on e-learning during COVID-19 pandemic.
Comparison (C)	Both types of study, i.e., with or without a comparison/control group were included.
Outcome (O)	Pharmacy students’ perceptions, satisfaction, attitude and/or opinions on e-learning during the COVID-19 pandemic.
Study design (S)	Any study (quantitative or qualitative) involving the collection of pharmacy students’ opinion or satisfaction or perception or attitude on e-learning during the COVID-19 pandemic.

**Table 2:** Main findings of the 23 selected studies.

Author, Year, Geographic Region, Database	Study Aim	Sample Size, Number of Pharmacy Students (Plus Other, If Applicable)	Methods	Findings	Discussion and Conclusion
**Studies Exclusively Involving Pharmacy Students**					
(Alghamdi et al., 2021) [29] Saudi Arabia and Egypt *PubMed*	*To explore pharmacy students’ perceptions and assess their attitude towards online education during the lockdown.*	241 out of 312 replied	Questionnaire (response rate 77%).	Students manifested an easy access to the technology, online skills, motivation, and overall favorable acceptance for online learning and examinations. Responses: “I think I learn more in online education than in face-to-face education” (36.1 agree or strongly agree); “I prefer online education to face-to-face education” (50.3% agree or strongly agree); “I feel more comfortable participating in online course discussions than in face-to-face course discussions” (70.2% agree or strongly agree) and “Online education requires more study time than face-to-face education” (44.4% agree or strongly agree).	Students have general acceptance for online education. However, only around half of the students preferred online than face-to-face learning.
(Alqurshi, 2020) [30] Saudi Arabia *PubMed*	*To investigate the effect emergency remote teaching has had on pharmacy education in Saudi* *Arabia, and to provide recommendations that may help set in place a contingency strategy.*	703	Questionnaires: two Likert scales (one for students and other for teachers).	Students from half of the studied colleges (9 out of 18 colleges), in general presented a good student satisfaction, while students from six colleges ranged between satisfied and unsatisfied students, and students from three colleges included some very unsatisfied students. The most explanatory variables of students’ satisfaction were, as follows: number and type of assessments, internet connection issues, limited interactions during lectures, and difficult to concentrate during virtual classrooms. Overall, 45% of students declared lack of guidance accompanied by unfamiliar methods of assessments. Concerns on the lack of student–student and student–teacher interactions: >35% of students.	A good student satisfaction only was achieved in half of the studied colleges. Recommendations: proactive learning strategies were purposed to overcome limitations of student–student and student–teacher interactions. A guide may help students to overcome constrains with assessments.
(Karattuthodi et al., 2022) [31] India and Saudi Arabia *DOAJ*	*To assess the quality of virtual education and students’ attitude and acceptance towards the new system during the second wave of COVID-19.*	482	Questionnaire.	Among other things, students declared: after lockdown, if online classes are offered as an option, I will choose it (Strongly Disagree or Disagree or Slightly Disagree = 53.5%); I prefer regular classes due to the following reasons [To get more knowledge] (Slightly Agree, Agree or Strongly agree = 93.1%); I prefer regular classes due to the following reasons (to discuss topics in the physical presence of teacher) (Slightly Agree, Agree or Strongly agree = 96%) or I prefer regular classes due to the following reasons (to conduct research/ practical works in the laboratory.) (Slightly Agree, Agree or Strongly agree = 96.9%).	The overall attitude and acceptance from the students were not satisfactory.
(Mendes et al., 2021) [32] Brazil *SciELO*	*To evaluate the satisfaction of the students studying Pharmacy with Emergency Remote Education, focusing on the learning process.*	401	Questionnaire; 401 out of 1025 (39.1%) students replied.	Students’ satisfaction with the Emergency Remote Education was on average 3.12 (scale 1 to 5): 37.9% of students (satisfied or totally satisfied, 4 or 5). Low satisfaction, regarding the quality of practical classes (3.06): 37.4% of students (satisfied or totally satisfied, 4 or 5).	The online format seems to require some improvements, especially regarding the practical classes. Slightly less than half of the pharmacy students declared being satisfied or totally satisfied, 4 or 5.
(Shawaqfeh et al., 2020) [33] United Arab Emirates *PubMed*	*To evaluate* *the pharmacy student distance online learning experience during the COVID-19 pandemic.*	309	Cross-sectional survey: questionnaire to evaluate students’ preparedness, attitude, and barriers (response rate of about 75%).	Average preparedness score: 32.8 ± 7.2 (Max 45). Average attitude score: 66.8 ± 16.6 (Max 105). Average barrier score: 43.6 ± 12.0 (Max 75). Students with positive attitude regarding e-learning: 49.2%. Students who have identified barriers regarding e-learning: 34%. Preparedness and attitudes scores significantly varied between different academic years (*p* < 0.05), with better results for the fourth-year students.	E-Learning was related to some issues, such as lack of preparedness, recognition of barriers regarding online learning or around half of the students manifesting poor attitudes. Finalists seem to manifest more favourable attitudes.
(Altwaijry et al., 2021) [34] Saudi Arabia *PubMed*	*To describe the experience of academic staff and students with distance education, during the COVID-19 pandemic, at a college of pharmacy in Saudi Arabia.*	Students (n = 223) and Academic Staff (n = 38)	A mixed-method approach: (1) survey to evaluate experiences of academic staff and students and (2) a focus group discussion to explore their experiences plus a five-point Likert scale. Response rate 78%.	Most students selected the option “true for me” (online education): “The amount of interactions with instructors”; “The amount of interactions with classmates”; “The distance learning process provides a personal experience that can be compared to the experience in the classroom”; ”Comfort to conduct homework’s and assignments during distance learning” and ”Comfort to study online for a longer period”. Most students selected the option “neutral” (online education): “The quality of interaction with instructors or classmates”; “Time management during distances learning period”; and “Academic achievements satisfaction during the distance education period”. Barriers and challenges: communication compared to face to face and health issues due to long time screen (students and staff).	Overall, participants showed a positive perception about online education. However, students pointed to diverse neutral domains and challenges in online learning.
(Liu et al., 2021) [35] Australia DOAJ	*To characterize pharmacy students’ challenges and strategies during COVID-19 curriculum changes.*	First-, second-, third-, and fourth-year pharmacy students (groups of 30 or 10–12 students) *	Collection of student written reflections, followed by codification. Five coders using NVivo 12 (March–May 2020).	Most coded challenges: ‘negative emotional response’ (frustration and anxiety were frequently reported) and ‘communication barrier during virtual learning’. The total number of references (students’ citations) for challenges were 589. Benefits (number of references = 68): Having satisfying placement Experiences; Less travel commuting; More family time; and Feeling valued and helpful during the pandemic. Most coded strategies were ‘using new technology’ and ‘time management’.	The identified challenges, benefits and strategies may help researchers and/or educators on achieving an adequate e-learning guidance. Both positive and negative experiences were identified, but the number of citations for challenges were much higher than the number of citations for benefits.
(Nagy et al., 2021) [36] Canada *PubMed*	*To understand* *how the learning of pharmacy students at the University of Alberta was impacted by the COVID-19* *pandemic.*	53 out of 397 pharmacy students replied	Questionnaire (response rate 13%). Open-ended questions: (1) how has the COVID-19pandemic situation affected your learning; (2) from a pharmacy and pharmacy school perspective, what have you learned since the COVID-19 pandemic began; and (3) from a personal perspective, what have you learned about yourself since the COVID-19 pandemic began?	Thematic analysis, with identification of two main topic: remote learning (learning environment, knowledge transfer, knowledge retention, assessment) and mental health (appreciation, stress, extroversion, motivation). Most students have a negative perception of online learning: “most students gave an initial statement that their learning was “impacted at all levels” and that the pandemic was “detrimental to [their] education.” Among the motives of students frustration were: “it takes longer to get through material” and “it was difficult to keep track of schoolwork.” Several students: home environment “loud and distracting” which was “not conducive of productivity.”	Most students have a negative perception of online learning, with two main motives being identified (e-learning and mental health status).
**Studies simultaneously involving pharmacy students and other healthcare students**					
(Alavudeen et al., 2021) [37] Saudi Arabia *PubMed*	*To evaluate health care students’ perception towards implementation* *of e-learning.*	Mixture Medicine (95, 37.4%); Pharmacy (125, 49.2%); Nursing (27, 10.6%); Others (7, 2.8%)	Questionnaire (April 2020 to July 2020).	Main barriers of students’ acceptance of e-learning: accessibility, inexperience, and unpreparedness. Pharmacy students (n = 125, 100%): COVID-19 affects my social and psychological wellbeing (No, 56.8%); E-learning improved the skills (No, 14.4%); E-learning has more limitations (No, 8.8%); E-learning is the future of education (No, 56%); E-learning is effective and helpful (No, 33.6%).	Overall, there was a limited student acceptance of e-learning. Pharmacy students identified both negative (e-learning have more limitations than attendance learning and will be not the future) and positive points (improvement of skills, effectiveness, and helpfulness of e-learning), with just a little more than half of students declaring no impact on social and psychological wellbeing.
(Almomani et al., 2021) [38] Jordan, Canada, Houston, and United Kingdom *PubMed*	*To study the influence of the COVID-19 pandemic and its associated quarantine on* *university students’ beliefs about online learning practice in Jordan.*	Mixture Pharmaceutical sciences (434,74.2%), General sciences (96, 16.4%), Engineering (47, 8%), and Literacy and humanities (7, 1.2%)	Questionnaire.	Students from second to fourth years were more prepared to deal with online learning than first year students. The majority of students (803%) declared that the quality of online education decreased when compared to school education. The opinion about the quantity of online education during COVID-19 pandemic decreased for 43.8% of students. Only 48.2% of students will register in online classes in the coming future. 61.5% of students classified as not fair the evaluation process used during the quarantine. Additionally, online exams were less preferred by 68% of students when compared to the in-campus exams.	E-learning during the pandemic have negatively impacted students’ beliefs and thoughts. Students were unsatisfied with quality and quantity of materials, provision of online exams, and the evaluation process.
(Al-Neklawy et al., 2022) [39] Egypt and Saudi Arabia *PubMed*	*To assess students’ recall, engagement,* *and satisfaction with the Blackboard (Bb) collaborate platform for online team-based learning (TBL),*	Mixture 306 Bachelor of Medicine, Bachelor of Surgery; 53 Nursing; 25 Doctor of Pharmacy, and 11 in Medical Laboratory Sciences Program	Online survey (the response rate varied between 26% and 73% per course type). All TBL sessions were recorded. Study implementation: randomization of teams, application of individual and team readiness assurance test, case applications, discussions intra and inter-team, instructions, peer evaluation and administration of the survey.	A high satisfaction with TBL was verified for all groups of students. Mean scores varied between 3.9 and 4.9 (maximum = 5) (e.g., “online TBL helped me increase my understanding of the course material” or “online TBL helped me meet the course objectives). All replies presented a statistically significant positive difference from the neutral mid-point response (*p* < 0.05)).	Blackboard platform for online team-based learning sessions was a successful learning tool for all groups of students.
(Chandrasiri and Weerakoon, 2021) [40] Sri Lanka *PubMed*	*To determine the perceptions of Allied Health Sciences* *undergraduates towards online learning during the COVID-19 outbreak.*	Mixture Radiography 170 (32.8%) Nursing 129 (24.9%) Medical Laboratory Sciences 94 (18.2%) Pharmacy 75 (14.5%) Physiotherapy 50 (9.7%)	Online questionnaire (the response rate varied between 9.7% and 73.2% per course type).	Perception score: mean 20.4 (4.0) (SD); maximum 27; Positive > 18, Neutral = 18, Negative < 18). 59.7% agreed that online learning is more comfortable to communicate than conventional Learning. 48.3% manifested a negative perception in relation to the offer of practical and clinical subjects online.	Most students presented a global positive perception of e-learning. However, almost half of the students manifested neutral or negative perceptions on online e-learning, with a negative perception score, regarding the administration of practical and clinical issues online.
(Rosillo and Montes, 2021) [41] Spain b-on	*To evaluate a gamification activity on mathematics, the Escape Room.*	Mixture Course 2020–2021, HyFlex System (Pharmacy = 23; Nursing = 13) Course 2019–2020, face-to-face (Pharmacy = 20; Nursing = 9) Course 2018–2019, face-to-face (Pharmacy = 20; Nursing = 21)	Questionnaires. A dual-mode approach using HyFlex System: Students may connect in face-to-face mode, online, or a mixture of both in the Escape Room.	Communication had improved more in the seminars carried out through the “Escape Room” than in the traditional seminars: 71% students. No difficulties in using ICT, or information and communications technology: 89% students. It found to be working more with the Escape room than in the traditional way: 76% students. For both pharmacy and nursing students, the valuations were not statistically significant different between the three courses and attendance was slightly higher in the course of 2020–2021 (HyFlex System).	The classroom environment, the students’ attendance to theseminars and the motivation improved in the the HyFlex System (course 2020-2021), with similar performances to the face-to-face training (courses of 2019–2020 and 2018–2019).
(Simon and Susamma, 2021) [42] Sultanate of Oman *DOAJ*	*To evaluate of the Evolving Student Experience* *During the Transition to Online Learning:Second-Language STEM Students*.	Mixture Medicine (793), Pharmacy (279), Engineering (2180) and School of Foundation Studies (497)	Administration of a questionnaire in two phases: Phase 1—1st April 2020 (response rate 31.2%) and Phase 2—21st April 2020 (response rate 15.4%). The second phase was optimized, regarding the outputs of the Phase 1.	Mobile access over PC was preferred by students. WhatsApp was more readily accepted. Synchronous instruction engaging students were more accepted than the asynchronous ones. Overall Effectiveness of Online Teaching-Learning: phase 1 (adequate, good, and very good = 41.7%). and phase 2 (adequate, good, and very good = 71.2%). Optimizations in the second phase: (a) interactive sessions, (b) better technology, and (c) the volume of available materials for students was reduced, since students considered the online learning hard.	Students seemed to learn at a slower pace and in a different way using online options. Online learning may be optimized and adjusted to the needs of students.
**Studies related to the involvement of pharmacy students in specific courses/ activities**					
(Reynolds et al., 2021) [43] USA *PubMed*	*To compare the effectiveness of distance-based experiential learning strategies to in-person experiential rotations, and explore student perceptions of knowledge, skills, and abilities gained through this adapted* *curriculum.*	6	An in-person course to provide in-person introductory experiential practice experiences was redesigned to be provided on-line. A 28-question survey at the end of the program. The six participants were from University of Colorado’s International-Trained PharmD students. The Mann–Whitney U test was utilized (pre- and post-course completion), which is a non-parametric test suitable for small samples.	Students agreed or strongly agreed that the overall distance course, the remote health system activities (e.g., Hospital Tour, Dispensing Operations, Practice Models), and the community activities (e.g., MyDispense tasks) valuable. MyDispense is a collaborative network of academic pharmacists who have formulated cases, content, and questions in this program. Students’ outcomes between both settings (in-person vs. online) were not statistically significant different for knowledge, skills, and abilities, but improved in online activities.	The redesigned course constitutes an alternative educational modality. However, students declared that they preferred live over online activities.
(Al-Alami et al., 2021) [7] Jordan *PubMed*	*To explore the effectiveness and student perspective of remote teaching of the theoretical anatomy and histology course.*	362 out 442 replied	Online-based validated questionnaire.	Around half of the students, and in some evaluated parameters slightly more, manifested positive perceptions. The less scored parameter was “the remotely-taught course contributed to a better understanding of the course content than I did before the lockdown” (40.8%). Both strengths (e.g., time flexibility) and weaknesses were identified (e.g., lack of face-to-face interaction, inadequate internet connectivity or other technical issues).	In general, pharmacy students’ perceptions regarding the effect of remote delivery of the theoretical anatomy and histology were positive, with a more restrictive output concerning the understanding of the content of the course (less than half). Some of the identified study weakness may be optimized in future training.
(Baumann-Birkbeck et al., 2021) [44,45] Australia and China b-on and *Cochrane Libray*	*To evaluate pharmacy students’ attitudes toward a virtual microbiology simulation.*	39 (completed the post-VUMIE™ (virtual microbiology simulation) survey) and 20 (completed the post-wet lab survey)	Surveys, a Likert scale (pre and post -intervention) plus collection of students’ comments. Comparison between a VUMIE and a traditional wet laboratory (lab). Response rates: around 50% at initial survey and around 25% at endpoint of survey.	The scores of the Likert scale were slightly higher for VUMIE than post-wet lab (overall, score VUMIE: mean score for the common rated items: 3.8 ± 0.78 VUMIE and 3.4 ± 0.76 wet laboratory (lab)). However, more students reported a specific preference for the wet lab rather than VUMIE, regarding the collection of students’ comments. VUMIE™ produced a slightly higher post-intervention mean scores (knowledge, skills, and confidence) when compared to the post-intervention mean scores of the wetlab.	Both activities were considered interesting and engaging. Study evidence was not sufficient to suggest a complete replacement of the traditional lab experience by VUMIE. The use of VUMIE previous to traditional wet laboratory (lab) work was suggested.
(Pearson et al., 2020) [46] United Kingdom b-on	*To explore the performance of online post-lecture chemistry* *crossword puzzles as revision aids prior to and during the COVID-19 pandemic.*	132 first-year and 120 second-year	Questionnaire. An online post lecture chemistry crossword puzzles.	80% of second-year students and just over 50% of first-year students found the crosswords helpful and would welcome more. In general, students agree with more crossword puzzles embedded within their online learning environment, with higher agreement scores for the second-year students. The three most scored online revision aids to help students were 1) instructional videos, 2) quizzes/puzzles and 3) practice questions.	Chemistry-themed online crossword puzzles were well-accepted by students, especially by the second-year students. Revision aids seems to be recommended in e-learning activities.
(Hussain et al., 2021) [47] USA b-on	*To examine pharmacy student readiness, reception, and performance in a communications* *course during the COVID-19 pandemic and to compare that with the performance of students who completed* *the same course in person the previous year.*	2019 (n = 25) and 2020 (n = 32)	Course 2019: face-to-face (15 lectures). Course 2020: online (16 lectures). Pre-course and post-course surveys were administered (pre survey n = 31 and post survey n = 26).	Student’s performance was not statistically significant different between both cohorts. Students’ preference for online education had grown by the end of the course, while face-to-face e-learning declined. The score for “the course should continue to be offered online and indicated that their online learning experience met their expectations for the course” was clearly favourable; M = 4.38 (agree = 4 and maximum 5 = strongly agree) (SD = 0.89). Students had previous e-learning experience.	Overall, student expectations with the online communications course seem to have been met. This study support e-learning after the COVID-19 pandemic.
(Elnaem et al., 2021) [6] Malaysia *PubMed*	*To investigate pharmacy students’ perceptions of various aspects of virtual objective structured clinical examinations (vOSCEs).*	231 out of 253 replied	Questionnaire. Response rate (91.3%).	Satisfied with vOSCE (53.2%). 49.7% of the students preferred to not have vOSCE in the future. The virtual OSCE was less stressful as compared to the conventional OSCE (36.8% strongly agree/agree). I feel that it would be more convenient to interact face to face with the examiners rather than a video call (53.7% strongly agree/agree).	Overall, only around half of the students were satisfied with vOSCE. vOSCE administration may need to be optimized in the future.
(Savage et al., 2021) [48] USA *PubMed*	*To explore student perceptions following implementation of a three-station remote* *OSCE administered during spring of 2020 and utilize the feedback to develop strategies for* *future remote OSCE implementations.*	157 (156 replied the questionnaire).	Two OSCE stations were implemented: (1) conducting a medication history interview on Day 1 and (2) presenting a patient case to a pharmacist preceptor and providing medication education to a patient on Day 2. Three open-text prompts about the remote OSCE experience were applied, as follows: (1) “I liked…”, (2) “I learned… ”, and (3) “I suggest … ”, which were administered the day after this remote experience. All replies were coded.	In general, students described this experience as positive and “applicable to their future pharmacy practice”. Diverse themes arose from this experiment. For instance, Logistics (n = 65, 41.7%), Differences In-person Versus Remote (n = 59, 37.8%), and Skill Development (n = 43, 27.6%). Among others, students classified as positive to receive materials ahead of time, clear instructions, to stay at home comfortably, or staying on schedule.	Students’ perception about the online OSCE activity was positive. OSCE is relevant to simulate telehealth activities, which will be more disseminated in the future. Students agree with the application of OSCE in the future.
(Sepp et al, 2021) [49] Estonia *PubMed*	*To compare the results of three face-to-face (2018–2019)* *and one electronically conducted (2021) OSCE tests, as well as students’ feedback on the content* *and organization of the tests.*	2018 (fourth-yearStudents: 12 (Auditorium); 2019 (fourth-year Students): 15 (Auditorium); 2019 (Assistant Pharmacists): 23 (Auditorium); 2021 (fourth-year Students): 28 (Zoom)	OSCE tests comprised diverse stations to simulate different themes (e.g., cough and sore; stuffy nose and allergy, dermatitis, etc., 3.5 min). Assessment of students at each station: establishing and ending contact; evaluation of symptoms, concomitant symptoms, comorbidities, and medications used; treatment recommendations; drug information; appropriate language use; and general health and well-being counseling. Student’s feedback was collected through a questionnaire.	Students were satisfied with the provision of OSCE test regardless of the environment (Auditorium vs. Zoom). The majority of students ranked OSCE as a “very good“ or “good“ learning method. Overall assessment of the OSCE test was not statistically significant different between face-to-face and Zoom OSCE.	The implemented zoom OSCE was feasible, effective, and students were satisfied with this practice. Overall assessment was similar between both Auditorium vs. Zoom OSCE.

* It was not possible to conclude about the total number of participants/students.

## Data Availability

The data presented in this study are available in the article.
